# First Implementation of a Point-of-Care Ultrasound Course in Undergraduate Medical Students in Peru: Mixed Methods Study

**DOI:** 10.2196/82717

**Published:** 2026-01-30

**Authors:** Otto Barnaby Guillén-López

**Affiliations:** 1 School of Medicine, Department of Clinical Medicine, Universidad Peruana Cayetano Heredia, Av. Honorio Delgado 430, San Martín de Porres, Lima, 15102, Peru, 51 3190000

**Keywords:** POCUS, point-of-care ultrasound, ultrasound, medical curriculum, curriculum, medical training, medical schools, competency-based education, undergraduate medical education

## Abstract

**Background:**

Point-of-care ultrasound (POCUS) is a test performed by physicians, as an adjunct to physical examination, to identify the presence or absence of specific clinical findings. This skill is not currently included in undergraduate medical education in Peru.

**Objective:**

This study aims to describe and evaluate the implementation of a POCUS course in undergraduate medical students.

**Methods:**

A pre-experimental study, without a control or comparison group, in which a pretest and posttest were used to evaluate the same group of students. A theoretical-practical POCUS course was designed and implemented for fifth-year medical students at the Universidad Peruana Cayetano Heredia in Lima (Peru) during late 2019 and early 2020. Their prior knowledge was assessed using a pretest consisting of 10 short-answer questions. At the end of the course, a posttest consisting of 9 different questions on ultrasound image analysis and recognition was administered, and the same 10 pretest questions were also re-evaluated. Satisfaction and perception of learning were also assessed through a survey. A descriptive analysis was performed, obtaining absolute and relative frequencies. The Wilcoxon test for related samples was used to evaluate the differences between the pretest and posttest.

**Results:**

A total of 26 students participated in the course, although only 19 completed the post-test (10 women and 9 men). The average pretest score before the course started was 4.8 (SD 2.2) points, indicating poor prior knowledge. This average increased to 18.5 (SD 1.6) points when they retested the pretest at the end of the course. The average posttest score was 12.2 (SD 3.3) points, which differed significantly from the initial pretest average (*P*<.001). Only 15 students responded to the satisfaction survey, with more than 50% reporting that they had fully acquired the ability to assess the inferior vena cava, bladder, free fluid in the thorax and abdomen, and right kidney. They also reported that the course met 97.5% of their prior expectations, but all considered the practical sessions with the ultrasound equipment to be essential. Although they considered that the best aspects of the course were learning how to use the ultrasound equipment and the small size of the groups, they suggested that the course could be improved by increasing its duration and the number of practical sessions, as well as by conducting the practical sessions with real patients presenting some type of pathology.

**Conclusions:**

We have successfully created a short theoretical and practical course on POCUS and have applied it for the first time to undergraduate medical students after their clinical rotations. This course has enabled them to perceive a significant improvement in their ability to recognize certain abdominal and pelvic organs and anatomical structures using ultrasound. This course can serve as a starting point for replicating POCUS teaching in medical schools across the country.

## Introduction

Ultrasonography is a noninvasive, portable, cost-effective, accurate, safe, efficient, and widely used clinical diagnostic tool [1], with few contraindications and adverse effects. In addition, ultrasound equipment is increasingly available to many physicians in the emergency and hospitalization areas of various hospitals in Peru and around the world.

Point-of-care ultrasound (POCUS), unlike a routine diagnostic ultrasound study performed by radiologists and ultrasound technologists, answers a clinical question posed by surgeons [2], emergency physicians [3], family physicians [4], and, in general, by any physician who treats a patient’s health problem using this procedure to solve it. POCUS is defined as an ultrasound examination provided and performed by the patient’s primary care physician, usually as an adjunct to the physical examination, to identify the presence or absence of a limited number of specific findings [5]. It has some synonyms such as clinical ultrasound, bedside ultrasound, or focused ultrasound [6].

As early as 2014, the American Academy of Emergency Medicine stated that “ultrasound should be integrated into the undergraduate medical education curriculum.” It even mentioned that there are numerous forms of introduction (teaching anatomy, physiology, physical examination, and procedures) and learning modalities, with 4 that report the best results: learning on “live” models rather than on simulators; e-learning based on cases or self-directed “podcasts” rather than lecture-based presentations; peer-to-peer practice rather than practice with the student “alone,” and a doctor-patient role-play model where they learn by participating as simulated patients [7]. However, to date, the skill of performing ultrasound studies is only taught in postgraduate programs and only in some medical specialties, and its teaching is not explicitly included in undergraduate programs at the main medical schools in Peru [8]. In their pilot study, Syrpeda et al [1] reported on the teaching and learning of some ultrasound skills and techniques in a small group of second-year medical students in practical sessions of 2 hours per week, promoting self-learning with minimal teaching guidance on the subject and achieving adequate levels of competence in obtaining and interpreting some ultrasound images.

We consider it necessary to introduce POCUS teaching into the undergraduate medical programs at our universities. This could also improve learning in certain subjects, such as anatomy and physiology, and certain skills and abilities, such as physical examination and diagnostic approach. Therefore, the objective of this research was to describe and evaluate the implementation of a POCUS course in a group of undergraduate medical students.

## Methods

### Overview

This was a pre-experimental study without a control or comparison group, as a pre and posttest was used to evaluate the same group of students. Quantitative and qualitative data were obtained. Students who were in their fifth year of medical school at the Universidad Peruana Cayetano Heredia in 2019 were included. The sample was convenience-based, consisting of those who voluntarily agreed to take the course through an invitation sent via institutional email. Students who started but did not fully complete the course were excluded, as well as those who had already received formal training in POCUS.

### Definitions

#### Knowledge of POCUS

This was measured using 2 assessments designed exclusively for this course. A pretest with 5 theoretical questions and 5 questions analyzing ultrasound images. A posttest with 9 questions, all analyzing images different from those used in the pretest. Each test had a minimum score of 0 and a maximum score of 20.

#### Perception of Achievement of Skill to Perform POCUS Studies

Students were asked about their perception of having achieved the ability to perform a POCUS study on a patient, and this was explored as a perceived percentage from 0% to 100%.

#### Satisfaction With the POCUS Course

To evaluate the impact on educational training at level 1 of the Kirkpatrick model [9], a satisfaction survey was conducted at the end of the course that evaluated the perceived percentage of usefulness of the course and some of its characteristics.

### Procedures and Techniques

The medical degree at Universidad Peruana Cayetano Heredia lasts 7 years, of which the first 3 are preclinical, the next 2 are clinical, and the last 2 are preprofessional internships in hospitals. To date, there is no specific POCUS course in any of the 7 years [10]. Therefore, the researcher designed a 3-week theoretical-practical course from scratch to teach the skill of using ultrasound equipment to perform POCUS studies in adults, identifying some images of importance in medical teaching and learning. This course was optional and voluntary, not part of the official curriculum, and was held between October 2019 and February 2020.

The theoretical part of the course was conducted exclusively through asynchronous individual reading of certain chapters of the ultrasound manual by García and Torres [11]: gallbladder, common bile duct, liver, kidneys, bladder, abdominal aorta, portal vein, inferior vena cava, and the E-FAST (Extended Focused Abdominal Sonography for Trauma) study. Specific learning objectives were drafted and sent to students via email prior to the practical sessions (Annex S1 in Multimedia Appendix 1 [11]). To ensure that the bibliography was read, a web-based formative pretest on the learning objectives was administered prior to each practical session, the results of which were not taken into account for this research.

The practical part was face-to-face and was led by the principal investigator, who is a specialist in internal medicine with training in POCUS since 2003 and more than 10 years of experience performing general ultrasound studies at the time of the study. Groups of 3 or 4 students were formed based on their own affinities. The practical sessions were carried out in three 2-hour sessions, during which the students performed ultrasounds based on what they had read the previous week. For these face-to-face practical sessions, each student took turns participating as “patients” (those who underwent the ultrasound study) and as “sonographers” (those who performed the ultrasound study), similar to that reported by Syrpeda et al [1] in order to learn normal ultrasound images. Each group was made up exclusively of either female or male students.

The researcher first performed each POCUS study on a volunteer student at the beginning of each session to show the other students what to do in practice. This also served as a role model for each of them, so they could then perform the same actions and maneuvers as the researcher, locating all the structures that were the subject of study in each session. The roles of sonographer and patient alternated between each student. The researcher stood next to the student at all times and guided them in the use of the transducer or corrected certain maneuvers, if necessary. The student had to show and point out each organ for the practice to be considered adequately performed. Each group had 4 practice sessions throughout the course. During the practical sessions, we were able to describe clinical cases in which abnormal findings could be found in the organ that the students were examining (eg, shock in the study of the inferior vena cava, acute renal failure in the study of the kidneys, cholecystitis or cholangitis in the evaluation of the gallbladder and bile ducts, etc). The cases were not standardized for each group and were used to contextualize the evaluation of each organ as a teaching strategy, so they are not shown in the study.

The ultrasound equipment used in the course was the Samsung SonoAceR3 model, available in the internal medicine hospitalization 1-II ward of the Hospital Nacional Arzobispo Loayza, and a 3.5 MHz convex transducer and a 7.5 MHz linear transducer were used, depending on the type of study performed. All course materials, notices, and communications were distributed through the Edmodo web-based classroom [12], which was available free of charge on the web until 2022. In this classroom, there were opportunities for question-and-answer forums to address any questions students had about what they were reading.

Although the study was conducted over 5 months, the specific duration of the course for each student was approximately 3 to 4 weeks, as the 3 practical sessions were held once or twice a week for each group, and the theoretical learning was asynchronous during the week prior to each practical session.

To determine students’ prior knowledge, the researcher designed an ad hoc pretest with 10 questions on theory and image analysis, the answers to which were to be developed and substantiated (Annex S2 in Multimedia Appendix 1 [11]). Multiple-choice questions were not used to eliminate the possibility of guessing, in case the student did not know or recognize what they were observing in the image they were asked about. If the student did not know the answer, they left it blank or wrote “I don’t know” and had a score of 0 on that question. The same pretest was also given to participating students to measure knowledge retention one week after the end of the course. Some questions from the pretest were:

- Which organic structure is the most hyperechoic, and which is the most anechoic when performing ultrasounds?
- Explain the purpose of performing the FAST protocol with ultrasound.
- Describe or list the 5 locations where ultrasound is performed in the extended FAST protocol.
- Mention the levels and cuts of protocolized visualization of the abdominal aorta.

To measure the achievement of learning objectives at the end of the course, a different posttest was designed (Annex S3 in Multimedia Appendix 1 [11]), with another 9 questions to be answered (short answer type), which only assessed the analysis and recognition of ultrasound images and were completely different from those used in the initial pretest.

**Figure 1.**
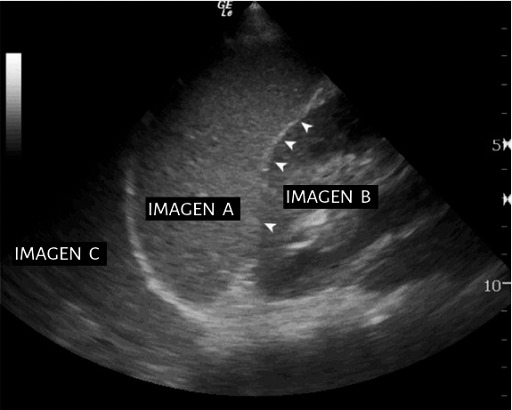
Image of question 2 from the posttest.

**Figure 2.**
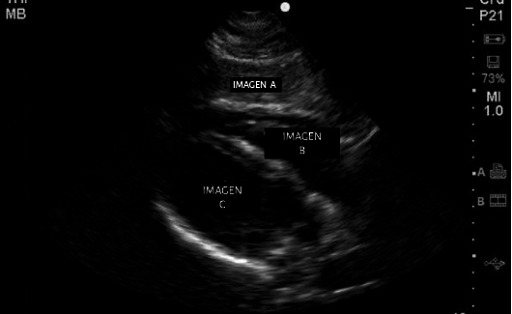
Image of question 3 from the posttest.

Some questions from the posttest were:

- In the image below (Figure 1), describe what each image corresponds to with a letter and arrowheads (there are 4 answers).
- In the following image (Figure 2), describe what each image corresponds to with a letter and what this projection is used for (there are 4 answers).

Neither the pretest nor the posttest underwent a rigorous instrument validation process, but they were tested in a pilot study conducted the previous year with similar students, who reported no problems understanding the questions or images. The tests were transcribed into Google Forms and then sent via email using a link, to be completed asynchronously by the students. All pretests and posttests were corrected by the same researcher following a scoring rubric (Annexes S2 and S3 in Multimedia Appendix 1 [11]). The maximum score for each was 20 points.

Likewise, after completing the posttest, students were sent a satisfaction survey (Annex S4 in Multimedia Appendix 1 [11]) to be completed via the SurveyMonkey platform. In this survey, the perceived usefulness of the course in general was evaluated, and students were asked to give a score from 1 to 10, which was then stratified as follows: essential (9‐10 points), very useful (6‐8 points), not very useful (1‐5 points), and not at all useful (0 points). The perception of the acquisition of different skills in ultrasound was also evaluated, and students were asked to give a score from 0% to 100%, which was stratified as follows: complete ability (100%), good ability (51%‐99%), little ability (1%‐50%), and no ability (0%). Students were also asked for open-ended opinions about the course, which are presented as they were written.

### Statistical Analysis Plan

A descriptive analysis was performed by obtaining the absolute and relative frequencies of the quantitative variables. The scores obtained in the pretest and posttest were subjected to calculation of averages with standard deviation and then to comparison of means using the Student t test. To evaluate the differences between pretest and posttest, the Wilcoxon test for related samples was used. A *P*≤.05 was considered significant. Some questions that required opinions are shown verbatim in quotation marks, but opinions that were repeated or only related to the specific content of the course were not included. Excel 2021 for Windows software was used for tabulation and descriptive analysis of the data.

### Ethical Considerations

The students authorized their participation by agreeing to take the course via email and by signing a written informed consent on the first day of practical sessions. The privacy and comfort of the students were respected by using an office, where only they were present in groups of 2, 3, or 4 people and the researcher; the office also had adequate ventilation and lighting. A stretcher and bedding were used to cover the parts of their bodies that were not undergoing ultrasound examination. The research did not involve any harm to any study participant, as it was an educational intervention. No student received any financial compensation for participating in the course, nor did they invest or spend any money.

This study was conducted after receiving approval from the Integrated Research, Science, and Technology Management Unit of the Faculties of Medicine, Stomatology, and Nursing, and from the Institutional Research Ethics Committee of the Universidad Peruana Cayetano Heredia with certificate number 480-20-19 dated August 27, 2019.

## Results

### Quantitative Results

**Figure 3.**
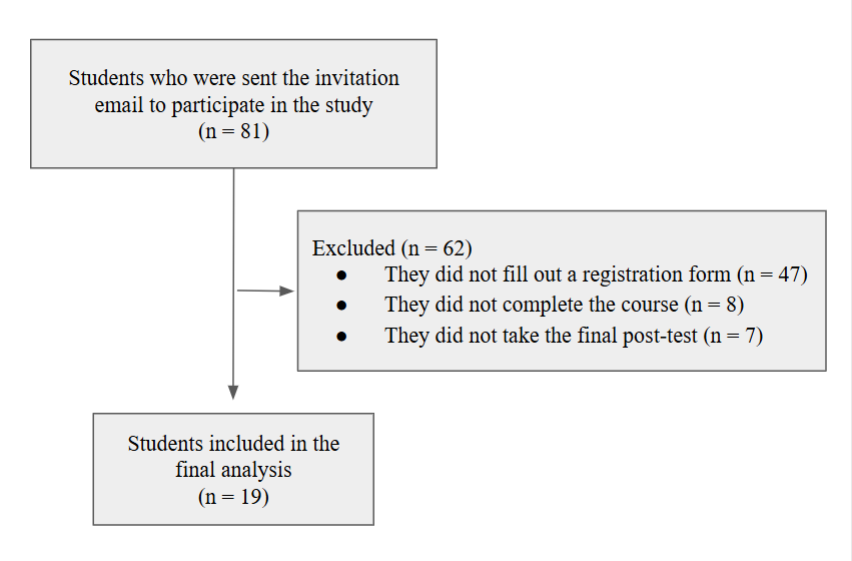
Flow chart of participant selection for the study.

An invitation to participate in the study was sent to 81 students; 34 enrolled, but only 26 participated in the course, although only 19 completed the posttest (10 women and 9 men). Figure 3 illustrates this. The mean age of these students was 22.2 (SD 0.8) years.

The 26 students took the pretest before the start of the course, with an average score of 4.3 (SD 2.2) points out of a maximum of 20, but only 19 (73%) students took the posttest at the end of the course. Table 1 shows the scores of these 19 students who took the 3 assessments (precourse pretest, end-of-course pretest, and posttest) between October 2019 and February 2020. The average posttest score was 12.2 (SD 3.3) points, and the average score obtained when retaking the same initial pretest was 18.5 (SD 1.6) points. However, there were 5 students (26.3%) who scored 10 points or less on the posttest, which made the final average lower than expected. One student even scored lower on the posttest than on the pretest at the beginning of the course. Nevertheless, it was evident that the students’ poor knowledge of POCUS before starting the course, with an average of 4.8 (SD 2.2) points, improved significantly in the posttest, where they obtained an average of 12.2 (SD 3.3) points, differences that were statistically significant (*P*<.001). In percentage terms, this meant an increase in precourse knowledge from 24% in the pretest to 61% in the posttest at the end of the course.

**Table 1.** Pretest and posttest notes from medical students, Universidad Peruana Cayetano Heredia, October 2019 to February 2020 (n=19).

Student	Pretest score basal	Pretest score at the end of the course	Posttest score
E1	5	17	13.5
E2	5	18.5	13.5
E3	3	20	12.5
E4	4.5	15	7.5
E5	6.5	16	12
E6	4	19	18
E7	6.5	20	15.5
E8	7.5	16	11.5
E9	4.5	19	16.7
E10	0	18	7.5
E11	5.5	19.5	10.5
E12	3	20	10
E13	7	19.5	13
E14	9	18.5	10.5
E15	4	19	12.7
E16	4.5	20	18
E17	2.5	17	9.5
E18	7.5	19	7
E19	2	20	12
Mean qualification (SD)	4.8 (2.2)	18.5 (1.6)^a^	12.2 (3.3)^b^

a *P*<.001 compared with the baseline pretest.

b *P*<.001 compared with the baseline pretest.

The course satisfaction survey was completed by only 15 students (57.7% response rate), who reported that the course met 97.5% of their prior expectations (range 90%‐100%).

Table 2 shows the perception of acquisition of the skills taught in the course. More than 50% of students reported that they had fully acquired 5 skills by the end of the course: assessing the size of the inferior vena cava (10/15, 66.7%), the volume and characteristics of the bladder (9/15, 60%), detecting free fluid in the thorax (8/15, 53.3%) and abdomen (8/15, 53.3%), and assessing the size and characteristics of the right kidney (8/15, 53.3%). The remaining skills obtained lower percentages. However, it is noteworthy that only a third or fewer of the students who responded to the survey considered that they had fully learned how to evaluate the common bile duct, spleen, gallbladder, and liver.

**Table 2.** Perception of acquisition of the skills taught in the course reported by medical students, Universidad Peruana Cayetano Heredia, October 2019 to February 2020 (n=15).

Perceived ability to evaluate each item	None or little, n (%)	Good, n (%)	Complete, n (%)	Total, n (%)
Size of the inferior vena cava in the study of a patient with hypotension	1 (6.7)	4 (26.7)	10 (66.7)	15 (100)
Bladder volume and characteristics	1 (6.7)	5 (33.3)	9 (60)	15 (100)
Free thoracic fluid	1 (6.7)	6 (40)	8 (53.3)	15 (100)
Free abdominal fluid	0 (0)	7 (46.7)	8 (53.3)	15 (100)
Size and characteristics of the right kidney	0 (0)	7 (46.7)	8 (53.3)	15 (100)
Pelvic free fluid	1 (6.7)	7 (46.7)	7 (46.7)	15 (100)
Free pericardial fluid	1 (6.7)	7 (46.7)	7 (46.7)	15 (100)
Size and characteristics of the left kidney	0 (0)	8 (53.3)	7 (46.7)	15 (100)
Portal vein size	1 (6.7)	7 (46.7)	7 (46.7)	15 (100)
Size and characteristics of the abdominal aorta	1 (6.7)	7 (46.7)	7 (46.7)	15 (100)
Size and characteristics of the spleen	0 (0)	10 (66.7)	5 (33.3)	15 (100)
Size of the common bile duct	2 (13.3)	8 (53.3)	5 (33.3)	15 (100)
Size and characteristics of the gallbladder	1 (6.7)	9 (60)	5 (33.3)	15 (100)
Size and characteristics of the liver	1 (6.7)	10 (66.7)	4 (26.7)	15 (100)

Table 3 shows the students’ perception of the usefulness of the different aspects of the course. All of them considered the teacher (15/15, 100%) and the practical sessions with the ultrasound equipment (15/15, 100%) to be essential. Meanwhile, the teaching methodology used in the course, with an emphasis on practice and asynchronous theoretical study with periodic evaluations, was considered essential by 93.3% (14/15) of students.

**Table 3.** Perception of the usefulness of the course and its materials reported by medical students, Universidad Peruana Cayetano Heredia, October 2019 to February 2020 (n=15).

Perceived usefulness of…	Not very useful, n (%)	Very useful, n (%)	Essential, n (%)
Course teacher	0 (0)	0 (0)	15 (100)
Practice with the ultrasound scanner	0 (0)	0 (0)	15 (100)
Course methodology	0 (0)	1 (6.7)	14 (93.3)
Learning objectives	0 (0)	2 (13.3)	13 (86.7)
Course manual	0 (0)	2 (13.3)	13 (86.7)
Course pretest	0 (0)	3 (20)	12 (80)
Course posttest	1 (6.7)	3 (20)	11 (73.3)
Web-based classroom	1 (6.7)	3 (20)	11 (73.3)
Course time	1 (6.7)	5 (33.3)	9 (60)

### Qualitative Results

Table 4 shows the open opinions of the students, who found that the best aspects of the course were putting theory into practice (5/15, 33.3%), learning to use the ultrasound equipment (3/15, 20%), and having few students per group (2/15, 13.3%).

**Table 4.** Textual responses to the question “Describe the best thing about the ultrasound course you took” from some medical students, Universidad Peruana Cayetano Heredia, October 2019 to February 2020.

Student^a^	Opinions
E1	“Learning how to use the machine, performing ultrasounds with my classmates, and seeing my organs.”
E2	"Using the ultrasound machine for the first time in my career, patiently and in a small group, gave me enough confidence to do the practice well.”
E3	“The content of the information is very accurate.”
E4	“Precise time for practice.”
E5	“To practice among ourselves and be able to see what’s normal.”
E7	“Put into practice what we had theoretically read.”
E8	“Learning to perform focused questions.”
E10	“Each chapter had specific objectives to accomplish.”
E11	"The teacher who always strives to ensure we effectively learn the basic concepts necessary for our careers, in addition to conveying knowledge well and seeking alternative learning methods, such as using a virtual classroom.”
E11	"Practicing with the ultrasound machine among students allowed us to learn what an ultrasound machine is like and how to use it, as well as to recognize anatomically normal structures without disease.”
E13	“Put into practice what you learned from the manual.”
E14	“Few students in each learning session.”
E14	“Do the practice based on clinical cases.”

a Some students wrote more than one different opinion. Others did not write any opinions at all.

Table 5 shows the students’ opinions on how to improve the course they took in this study. These were mainly related to having more course time and more practical sessions with the ultrasound equipment (5/15, 33.3%) and performing practical sessions on real patients with pathologies (6/15, 40%). This suggests that although students consider the course to be very valuable, it could be improved by increasing the duration of the course and the number of teaching assistants.

**Table 5.** Textual responses to the question “Describe suggestions you consider to improve learning in the ultrasound course you took” from some medical students, Universidad Peruana Cayetano Heredia, October 2019 to February 2020.

Student^a^	Suggestions for improvement
E1	“More topics”
E2	"When we couldn’t find a position for the ultrasound during practice, sometimes the doctor would help us guide the transducer, but even then, I often felt like I didn’t know where it was pointing. It would have also helped to hear an anatomical reference or a verbal tip on how to locate it at that moment to remind me where to look”
E2	"Shorten the time between practices. A consistent week-to-week rhythm of these practices would seem ideal to consolidate knowledge and practice with the ultrasound”
E2	“See cases of pathologies”
E3	“More practice time”
E4	“Clinical cases in each practice, including echocardiographic studies”
E5	“It would be possible to see pathologies in real patients using ultrasound”
E7	“Having the opportunity to perform ultrasound on real patients”
E7	“Conduct more sessions on other uses of ultrasound”
E8	“Perhaps we could also practice it on a patient with a pathology related to the corresponding topic”
E9	“I think one more session would be beneficial so as not to cover so many topics in the second session”
E11	"With regard to the learning objectives, perhaps it would have been more appropriate to also mention that we review topics such as cases of biliary colic, hypotensive patients, or some cases that were seen in the pretest, because although we should have that knowledge from our previous years, it would have been important to mention them so that students could review and have an idea of how to relate the ultrasound manual to clinical aspects”
E11	"Although the central theme of the course is learning how to use ultrasound and asking focused questions, it would have been more interesting to have given us more examples so that we could ask more focused questions, because otherwise the course would only focus on learning ultrasound, but not POCUS, which would involve seeing patients or identifying pathological findings”
E11	“Time will always be a difficulty that is not necessarily the fault of the students or the teacher”
E12	“I wish I could have more days to practice”
E14	“Maybe I can practice with a patient with a certain pathology after having practiced”
E15	“I would like to be able to practice on patients with pathology”

a Some students wrote more than one different suggestion. Others did not write any opinions at all.

## Discussion

### Principal Findings

Although by 2023, 18 of 53 (31%) medical schools in the European Union [13] and, by 2020, 56 of 122 (56%) undergraduate medical schools in North America and other countries included POCUS instruction in their curricula, only 3 were from South America, specifically Brazil [14]. The theoretical and practical course that was the subject of our study was conducted between October 2019 and January 2020, at a time when there was little or no experience of using POCUS as a learning methodology in undergraduate medical studies in Peru [8]. Its design and subsequent application in a group of volunteer students before they began their preprofessional internships in hospitals showed favorable results, both in learning how to use ultrasound equipment and in knowledge of theoretical aspects and recognizing normal images in ultrasound studies in some areas of human medicine. This was reflected in the results of the postcourse evaluation, which showed significant differences with those of the precourse evaluation, indicating adequate acquisition of the knowledge and skills taught.

Our course was created from scratch following the 6 steps described by Kern and reported by Rivera Mercado et al [15], using the information we had previously obtained on the learning needs of medical students in 2018 [8]. The content and learning objectives of our course partially coincide with those used in a medical school in a rural area of Idaho, United States, in 2022 [16], and, although the latter was implemented in first-year medical students and lasted longer than ours, it also demonstrated a significant increase in their knowledge of POCUS. Meanwhile, in 2023, POCUS was taught from the first to the sixth year of medical school and was mandatory in some institutions in the European Union. In some of these institutions, it was even taught longitudinally over several years, although in others, it was offered transversally in subjects such as anatomy, cardiology, gastroenterology, or pulmonology [13].

At Jichi Medical University in Japan, where the medical degree lasts 6 years, ultrasound education has been provided to medical students since 1978 and is taught from the second to the final year of the degree in different courses. The 2021 curriculum also includes ultrasound training content in courses on cardiology, gastroenterology, nephrology, orthopedics, obstetrics, and emergency medicine. However, it had not yet been used for formal teaching of basic sciences or physical examination [17].

By 2022, a study in Denmark reported that 95.7% of physicians had received formal ultrasound training during medical school, although only 71.7% of their training was practical, and only 20.7% were formally evaluated in ultrasound during their medical studies [18]. The content most commonly included in the ultrasound training of these medical students was FAST, heart, lung, peripheral venous access, gallbladder, bladder, kidney, abdominal aorta, and liver, which were very similar to those taught in our course.

### Comparison With Prior Work

We found only 2 studies in Latin America on POCUS teaching in undergraduate medical education in São Paulo, Brazil, and basically on E-FAST. One was developed at the University of São Paulo in 2017, where a 50-minute ultrasound course was given to 37 medical students, most of whom were in their first 4 years of study, and improvements in knowledge were found, even up to 3 months after the course ended [19]. However, this study did not evaluate the learning of practical skills. The other was conducted with 66 sixth-year medical students at Santo Amaro University in 2015 in a 5-hour program of theoretical and practical sessions, and it was shown that students improved their knowledge of E-FAST, although this was not maintained sufficiently after 3 months. However, in this study, a practical assessment was conducted at the end of the course, and it was found that 81.8% of students demonstrated skills in acquiring ultrasound images, especially of the hepatorenal and pulmonary windows [20].

In our study, although we were unable to evaluate ultrasound diagnostic imaging skills in a practical and objective manner, more than 67% of our students perceived that they had fully acquired the skills in assessing the inferior vena cava and bladder, and more than 53% of them felt that they had acquired the skills to detect pleural and ascites fluid and the right kidney after taking our course. Similarly, a study in Saudi Arabia in 2023 reported that most final-year medical students perceived that they had acquired adequate skills to detect free abdominal fluid, hepatomegaly, deep vein thrombosis, and thyroid masses, but at least 31.5% of them had received some prior POCUS training at their faculty [21]. Meanwhile, in 2021 and 2022, another theoretical-practical course for second-year medical students also reported full acquisition of skills in assessing the liver and right kidney, Morrison’s space, the subxiphoid and long parasternal views of the heart, and the pleural line in Indianapolis, United States [22]. However, in the latter study, almost 40% of students also had some prior experience with POCUS. In our study, all students reported that they started their ultrasound training from scratch, although, as they were students finishing their fifth year of medical school, they may have been able to use their prior knowledge of anatomy and physiology to achieve better learning outcomes.

Meanwhile, a study conducted in Germany at the same time as ours also demonstrated a significant improvement in learning after a POCUS course focused on the liver, gallbladder, spleen, kidney, pancreas, urinary bladder, and spaces for evaluating free fluid or pleural effusion, as well as the thyroid and vascular structures, in fourth- and fifth-year medical students [23]. On the other hand, good learning in the evaluation of the spleen, right kidney, and urinary bladder was also observed in another study, which was even conducted with instructors who were peer students with experience in POCUS, and these skills could be maintained for up to 4 months after instruction of only one day of theory and one day of practice at different times [24].

The greatest contribution of our study was to show that it is possible to design a POCUS course for students in the clinical years of undergraduate medical school, implement it in a practical way in a few weeks with a single qualified instructor, and evaluate the knowledge acquired by students not in a theoretical way, but analytically, through exams requiring short answers about the analysis of POCUS images. In this regard, most studies on the implementation of POCUS courses in medicine are carried out for postgraduate students in different medical specialties (emergency medicine, internal medicine, critical care, anesthesiology, rheumatology, nephrology, pediatrics, etc) and are evaluated with multiple-choice exams, which only measure memorization [25]. However, some studies conducted in other countries, particularly the United States and Canada, have already implemented POCUS and proposed its inclusion in the medical school curriculum beyond the preclinical years (anatomy and physiology learning). For example, in Utah, United States, in 2022, a pilot course of self-directed learning was conducted with medical students in their clinical rotation with internal medicine inpatients, and they mentioned that in addition to being able to obtain adequate ultrasound images of the heart and lungs, they were able to improve their clinical reasoning, learning of pathophysiology, and medical decision-making and care for patients with cardiorespiratory pathologies [26].

Since 2021, the Medical University of South Carolina has implemented a 3-year POCUS program for its undergraduate students starting in their second year, teaching various related topics (pulmonary, cardiac, and abdominal ultrasound, studies for deep vein thrombosis) using educational videos, high-fidelity simulators, and hands-on workshops [27]. This program improved students’ overall knowledge of POCUS from 50% before the program to 67% after it, which was similar to the 61% average posttest scores achieved in our course. Although our students started with an average pretest knowledge score of 24% at the beginning of the course, the questions on our posttest were completely different from those on the pretest, and images were also used for analysis in their assessment.

At the Mayo Clinic in Minnesota, United States, an elective POCUS course was offered to medical students prior to their clinical years between 2021 and 2023. In this course, which was conducted theoretically through individual video viewing and 3 in-person practice sessions, students learned basic knowledge of POCUS, E-FAST, abdomen, etc, topics similar to those in our course. These students improved their precourse test scores from an average of 56.3% to 73.3% on the postcourse test [28]. However, the practical sessions in this course lasted 9 hours per student, while in ours they lasted 6 hours per student.

At the University of Connecticut, United States, a sustainable, vertically integrated 4-year POCUS curriculum was created, which they began to develop in 2016. During the 2021‐2022 academic year, more than 400 medical students who took this curriculum were evaluated using a objective structured clinical exam, and it was found that the students improved their basic knowledge of ultrasound, as well as their orientation, selection of transducers and modes, and were even able to independently acquire and identify 11 of 18 anatomical structures using POCUS [29]. This demonstrates that POCUS teaching can be included not only as a stand-alone course in medical school but also longitudinally throughout all years of study.

The expectations of the students regarding our course were met in more than 97% of them, probably due to the predominance of the practical methodology of the course and teaching in small groups, which is consistent with other similar studies [16,30,31]. In fact, the main objectives of this course were captured by 2 students who commented: “Practicing with the ultrasound machine among students allowed us to learn what an ultrasound machine is like and how to use it, as well as to recognize anatomically normal structures without disease” and “Using the ultrasound machine for the first time in my career with patience and in a small group of people gave me enough confidence to do the practice well.” This improvement in confidence in the use of ultrasound was also reported by other authors in Germany, Canada, and Poland, even with only one day of instruction in POCUS [32-34].

However, there were still areas for improvement in our course, such as those related to performing ultrasound studies on patients with pathologies and, especially, having more practice time with the ultrasound equipment, as reported in other studies [18,32,33]. All suggestions for improvement could be achieved in the future by obtaining more POCUS-trained teachers and more ultrasound equipment, if teaching can be implemented in undergraduate programs and funding and logistical support can be obtained. In other countries, the use of peer teaching by students as POCUS instructors [29,31,35], and low- and high-fidelity simulation strategies have been proposed, each with several advantages, which could even complement each other to contribute to or develop other skills, such as empathy, communication, and teamwork, among others [36]. Most importantly, regardless of the duration of our course or its future voluntary or mandatory implementation in the medical school, after learning these skills, each student should continue to use POCUS in every area of the rest of their undergraduate studies and their future professional career.

### Limitations

We group the limitations of our study into several types. First, those related to the development of the course itself, as the number of students who completed the course was lower than initially expected when the call for applications was made, which may have been related to the fact that they were taking regular courses that they had to pass at that time at the university and were unable to participate or complete the entire course. Other students had to travel abroad at the end of 2019, despite initially enrolling in the course. This meant that the final number of students who took the posttest was less than 20. Although this does not allow us to generalize the results obtained in our study, the large difference found between the pre- and postcourse assessments in almost all students could indicate that the course was indeed useful for learning how to perform POCUS. Another limitation was that the students voluntarily enrolled in the POCUS course, which could have positively influenced the learning of new concepts, as they were highly motivated to learn and faced a selection bias.

A second type of limitation is related to the assessment methods. On the one hand, learning was measured with image recognition and knowledge tests shortly after the course was completed. It was not possible to conduct a follow-up assessment a few months after the course ended due to circumstances arising from the COVID-19 pandemic and because contact with the students was lost, as communication with them was only through institutional email, access to which is lost upon graduation from the university. Nowadays, follow-up evaluations of the course can be carried out after a few months, as the skills and knowledge learned in the course may be forgotten if they are not continued to be practiced. On the other hand, there was no time, teachers, or resources to design and carry out practical evaluations to determine the acquisition of operational skills in the use of ultrasound equipment and transducer management to obtain images of the organs to be evaluated.

Subsequent research may also include assessments of level 3 of Miller’s pyramid, such as objective structured clinical evaluations, as performed in other countries, so that students can “show how” they perform the ultrasound study [37]. Rubrics can also be used to review video clips of the students themselves performing an ultrasound examination, with subsequent feedback [16] or direct observation of procedural skills [32,36].

Another type of limitation could be related to biases in the qualitative part of the study, as there could have been response bias, since students, even though the responses were anonymous, could have expressed favorable opinions about the course in order to look good in front of the professor or to acknowledge his effort and dedication. There could also have been a possibility that students who completed the course but did not respond to the satisfaction survey had less favorable opinions. However, this risk may have been minimal because the questions were open-ended, specific, and precise (Annex S4 in Multimedia Appendix 1 [11]), and because some students also described several areas for improvement in the course. Nevertheless, both types of opinions are useful to take into account for future similar research and subsequent applications of the course.

### Other Relevant Aspects of the POCUS Course Implemented

Nevertheless, there are several relevant aspects to our research. One was that no real patients were needed for the practical sessions, as the students were able to perform the POCUS studies on each other. This may have created a more comfortable learning environment than in a hospital ward or health center office with real patients. Furthermore, although many medical schools in other countries include POCUS from the first year of the degree in anatomy, physiology, or physical examination courses [14], our study was conducted prior to the students’ preprofessional practices, when they had already taken medical clinical courses, which may have positively influenced the learning of POCUS skills by using some prior knowledge of anatomy and physiology. However, over time, it could be implemented earlier in the degree program once certain barriers described above have been overcome, such as the lack of trained teachers, the lack of space in the current curriculum, or the lack of ultrasound equipment [14]. Another unique aspect of our study is that, unlike almost all studies on POCUS teaching in undergraduate medical education, we conducted assessments with short-answer, open-ended, or essay questions. By not using multiple-choice questions in our course assessments, we reduced the possibility of students guessing the answer by “recognizing” the correct answer rather than remembering or generating it, or using exam strategies to “eliminate incorrect alternatives” without requiring in-depth knowledge of the subject [38]. Several authors have described and demonstrated that short-answer questions have similar reliability and discrimination as multiple-choice questions [38-40]. A final important point was that at the end of the course, we only assessed the interpretation of ultrasound images and not the theoretical aspects of the subject, which is required to be competent in medical ultrasound and had been included by other authors [30,36]. This could indicate that students had also mastered the modality of acquiring some ultrasound images.

Something we noticed during the practical sessions was that some students had deficiencies in their theoretical knowledge of abdominal and vascular anatomy, and these were precisely the students who had the most difficulty recognizing some images and locating anatomical structures during abdominal ultrasound examinations. Therefore, we suggest that, if this proposed POCUS course is implemented in the undergraduate medical curriculum, it should be taken after sufficient knowledge of anatomy has been acquired, at least of the abdomen, urinary tract, and pelvis. Alternatively, a prior review of the anatomy of the organs and systems that will be covered in this course should be included.

Finally, we sought to develop several practical skills in students beyond the course: (1) the ability to apply the theoretical and practical knowledge acquired in the course to real situations, since the following year they would be treating patients in their hospital rotations, (2) the ability to solve complex cases or problems in a simulated manner, since when conducting the practical sessions with the ultrasound equipment, we told the students about clinical cases in which they might encounter an abnormal finding in the organ they were exploring, and (3) reflection on continuing to learn how to use ultrasound equipment wherever they are rotating or working in order to further develop the skills learned in this course. We even thought that in this course, we could have recruited some students to act as teachers or instructors and thus replicate the course in other groups of students later on. However, the circumstances of the pandemic prevented us from achieving this.

### Conclusions

Our theoretical-practical POCUS course, conducted in small groups of undergraduate medical students after their clinical rotations, demonstrated that it was able to achieve a significant improvement in their recognition of certain organs and anatomical structures using ultrasonography. Likewise, the students who participated in the course found it very useful to learn the skill of performing POCUS to identify and interpret some normal findings in abdominal and pelvic anatomy using ultrasonography. Therefore, we suggest that it can be progressively implemented in undergraduate medical programs in Peru, as it already exists in other countries.

## Supplementary material

10.2196/82717 Multimedia Appendix 1 Course design, pretest, posttest, and satisfaction survey.

## Abbreviations

E-FAST: Extended Focused Abdominal Sonography for Trauma
POCUS: point-of-care ultrasound
