# Supplementary material for: First Implementation of a Point-of-Care Ultrasound Course in Undergraduate Medical Students in Peru: Mixed Methods Study
Source: JMIR Form Res. 2026 Jan 30;10:e82717. doi: 10.2196/82717 (PMC12858223; doi:10.2196/82717)
Supplement: Multimedia Appendix 1 [file formative-v10-e82717-s001.docx]

# SUPPLEMENTARY MATERIAL

**Annex S1: Course design**

| **PURPOSE:**  The course is theoretical and practical and aims to enable students to develop skills in performing ultrasound examinations to improve their diagnostic planning and/or management skills for patients with certain medical and surgical conditions.  **LEARNING OBJECTIVES:**  At the end of the course, **fifth or sixth-year** medical students will be able to:   - Use in a basic way a portable ultrasound equipment to obtain two-dimensional images of some organs of the human body. - Perform and analyze ultrasounds using the E-FAST protocol - Perform and analyze ultrasounds to detect free fluid in the pleura, pericardium, and peritoneum in certain medical and surgical conditions. - Perform and analyze ultrasounds to evaluate the kidneys and bladder. - Perform and analyze ultrasounds to detect the gallbladder and common bile duct. - Perform and analyze ultrasounds to detect the portal vein, liver, and spleen. - Perform and analyze ultrasounds to measure the diameter of the abdominal aorta and iliac arteries. - Perform and analyze ultrasounds of the femoral and popliteal veins to detect blood clots in a patient with suspected deep vein thrombosis. - Perform and analyze ultrasounds to evaluate the inferior vena cava to estimate the value of central venous pressure - Perform and analyze ultrasounds to evaluate the internal jugular vein to estimate the value of central venous pressure   **METHODOLOGY**  The practical part of the course will consist of students using a portable ultrasound machine, alternating between performing the ultrasound themselves (“doctor”) and acting as a model for the ultrasound (“patient”), with guidance from a teacher who is an expert in point-of-care ultrasound (POCUS). The theoretical part will be taught through independent reading of some chapters of a manual on ultrasound by García and Torres (1)  **PLAN AND IMPLEMENTATION**  ***Teaching modality:***   - Independent reading of chapters from an ultrasound manual. - Viewing some videos about ultrasound in a web-based classroom. - Discussions in web-based forums about the videos watched. - Workshop in small groups with practical activities among peers, led by a teacher with basic knowledge of ultrasound. - Practical sessions between the same students alternating as “sonographers” and “patients”.   ***Topics:***   - Using the Samsung SonoAce R3 ultrasound system - E-FAST protocol in the study of patients with potential multiple trauma - Ultrasound evaluation of kidneys and bladder in a normal patient - Ultrasound evaluation of the liver, portal vein, and spleen in a normal patient or a patient with liver disease - Ultrasound evaluation of the gallbladder and common bile duct in a normal patient or with biliary pathology - Ultrasonographic evaluation of the abdominal aorta and common iliac arteries in a normal patient - Ultrasonographic evaluation of the common femoral vein and popliteal vein in a normal patient or with deep vein thrombosis - Ultrasound evaluation of the inferior vena cava in a normal patient   **LEARNING EVALUATION PLAN:**   - Pretest before the course. - Posttest of ultrasound image analysis at the end of the course. - Practical assessment using a checklist (optional) |
| --- |

**Annex S2: Pretest and Correction Guidelines**

| 1. Which organic structure is the most hyperechoic and which is the most anechogenic when performing ultrasounds? 2. What is the purpose of performing the FAST protocol with ultrasound? 3. Describe or list the 5 locations where ultrasound is performed in the extended FAST protocol. 4. Mention the levels and sections of the protocolized visualization of the abdominal aorta 5. At what size is the diameter of the abdominal aorta and iliac arteries abnormal? 6. Describe how bladder urine volume is estimated using ultrasound. And what is the value in milliliters (mL) that indicates a patient has urinary retention? 7. In the following image 7, write what each image or arrow corresponds to:   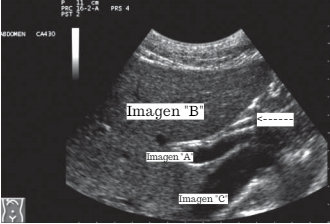   1. In image 8, write what each image corresponds to:   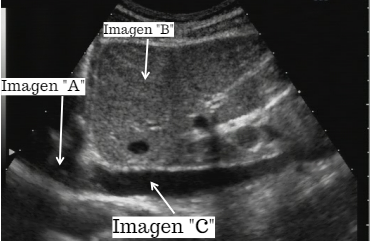   1. In image 9, write what each image and arrow corresponds to:   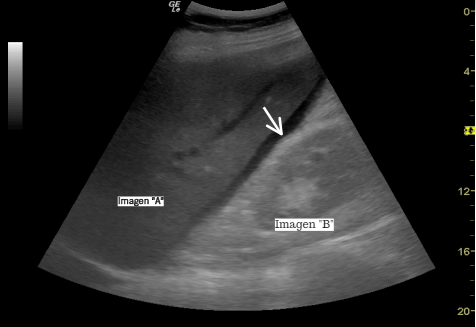   1. Approximately how many centimeters of measurement and percentage of collapse during inspiration of the inferior vena cava correspond to a central venous pressure (CVP) of 10-15 mmHg?   *Correct answers:*   1. Bone (hyperechogenic), fluid (anechogenic) 2. Morrison's pouch (Hepatorrenal recess), splenorenal recess, pelvis (rectovesical pouch in men or Pouch of Douglas in women), cardiac view (subcostal or subxiphoid), and lateral thorax view (pleural effusion). 3. Evaluation of the polytraumatized patient to determine hemopericardium or intra-abdominal bleeding 4. Proximal transverse, medial transverse, distal transverse, distal transverse at the level of the bifurcation in the iliac arteries, and longitudinal of the abdominal aorta along its entire length. 5. Aorta: 3 cm, Iliac: 1.5 cm 6. 0.5 x length x width x height = volume (in mL). Urinary retention occurs when more than 150 ml of urine remains. 7. A: Portal vein, B: Liver, C: Inferior vena cava, Arrow: Common bile duct 8. A. Right atrium, B. Liver, C. Inferior vena cava. 9. A. Liver, B. Right kidney, Arrow: free fluid in hepatorenal space (Morrison's fossa). 10. 1.5 - 2.5 cm and collapse less than 50% |
| --- |

**Annex S3: Posttest and correction guidelines**

| 1. In the image, write down what each image corresponds to with a letter and the structure measured with the number “1” (there are 5 answers): 3 points   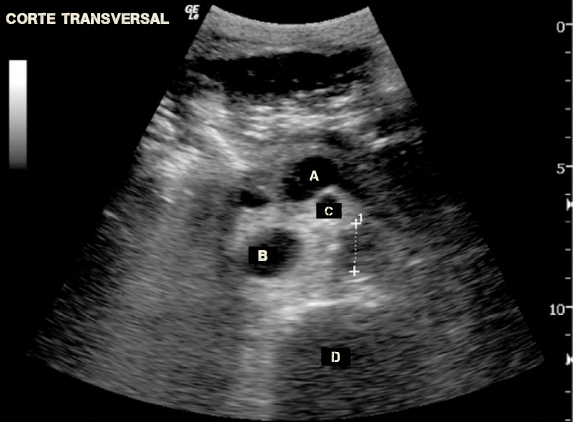   1. In this image, write down what each image marked with letters and arrows corresponds to. (there are 4 answers): 2 points   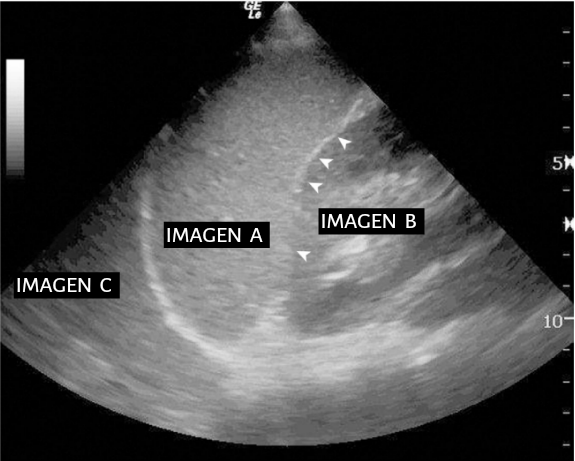   1. In this image, Write down what each image corresponds to and what this projection is used for (there are 4 answers): 2 points   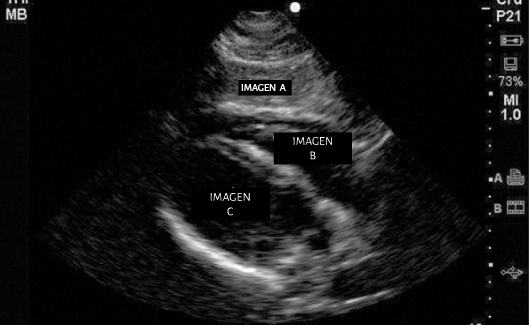   1. In this image, write down what each image marked with a letter corresponds to and what type of ultrasound view it is (there are 3 answers): 1 point   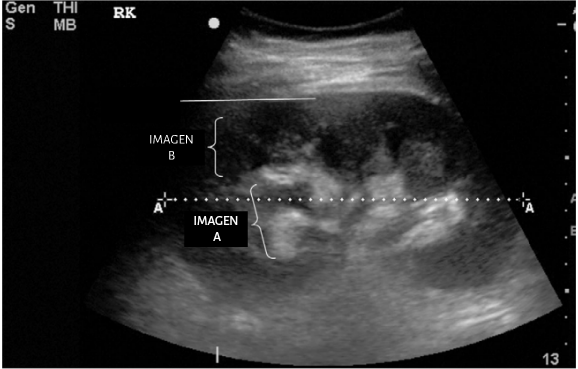   1. In this image, write down what each image marked with a NUMBER corresponds to (there are 6 answers): 3 points   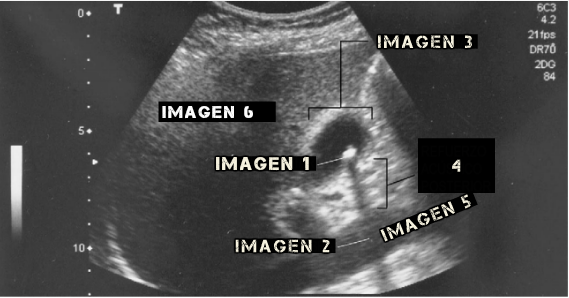   1. In this image, write down what each image marked with a letter corresponds to and what this ultrasound view is used for in the FAST study. (there are 3 answers): 1 point   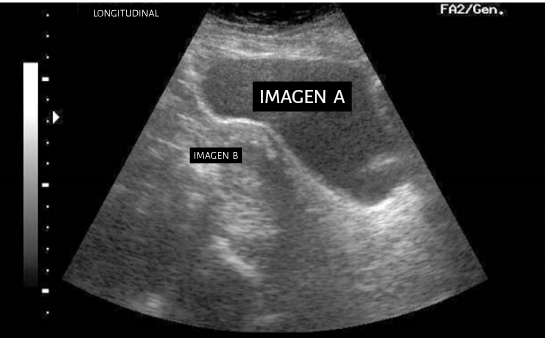   1. In this image, calculate the percentage of collapsibility (write the formula) and estimate the central venous pressure (CVP) accordingly. Briefly explain your findings in the context of a patient with arterial hypotension. 3 points   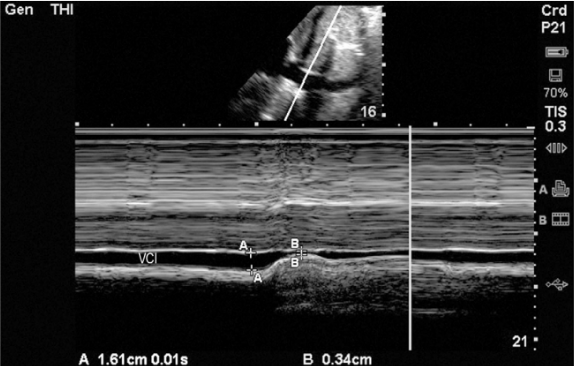   1. In this image, calculate the percentage of collapsibility (write the formula) and estimate the central venous pressure (CVP) accordingly. Briefly explain your findings in the context of a patient with arterial hypotension. 3 points   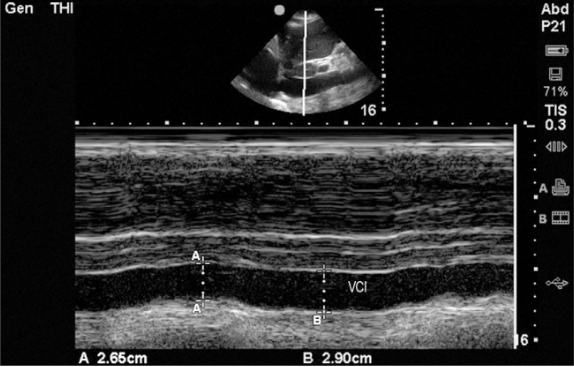   1. In this image, write down what each image marked with a letter corresponds to (there are 3 answers): 2 points   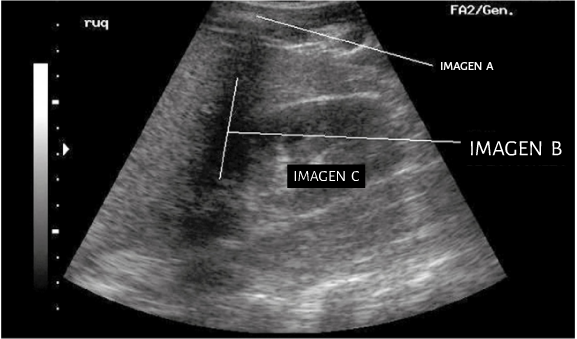  *Correct answers:*   1. D: acoustic shadow of the vertebral body, B. inferior vena cava (IVC), C. superior mesenteric artery, A. splenic vein 2. A. Spleen, B. Left kidney, C. Left lung (actually a mirror image of the spleen), Arrows: splenorenal recess (represented by Gerota's fascia which is hyperechoic). 3. A. Liver (used as a window to view the heart), B. Right ventricle (located more anteriorly), C. Left ventricle. The projection is used to determine if there is fluid in the pericardial space (which seems to exist). 4. A: renal sinus, B: renal parenchyma, longitudinal view of the kidney 5. 1. Gallbladder stone, 2. Acoustic shadow of the stone (assuming that number 5 refers to the IVC), 3. Thickened gallbladder wall, 4. Posterior reinforcement of the gallbladder wall (hyperechoic) or acoustic shadow of the stone (anechoic), 5. Inferior vena cava (IVC), 6. Liver 6. A. Bladder, B. uterus. This view is used to determine if there is free fluid (ie blood) in the steepest part of the pelvis. 7. Collapsibility = (2.9 - 2.65)/2.9 x 100% = 8.62%, approximate CVP would be: > 2.5 cm with minimum collapsibility: 15-20 mmHg CVP. In the context of a case with arterial hypotension, this would not be hypovolemic shock. 8. Collapsibility = (1.61 - 0.34)/1.61 x 100% = 78.88%, approximate CVP would be: < 1.5 cm with collapsibility > 50%: 0-5 mmHg, 1.5-2.5 cm with collapsibility > 50%: 5-10 mmHg. Conclusion: hypovolemia, volume must be replaced. 9. A. rib; B. acoustic shadow of the rib, C. kidney. |
| --- |

**Annex S4: Satisfaction survey**

| 1. Please indicate from 0 to 10 the usefulness of the following in relation to the 3 weeks of the ultrasound course you took  - General course - Course methodology - Learning objectives - Course teacher - Course manual - Course time - Course pretest - Course posttest - Ultrasound equipment - Practice with the ultrasound scanner  1. Please indicate the percentage of your current capacity to evaluate the following cases using an ultrasound: 0%, 1-50%, 51-99%, 100%  - Free thoracic fluid - Free abdominal or pelvic fluid - Free pericardial fluid - Size and consistency of the liver - Size and consistency of the spleen - Size and characteristics of the right kidney - Size and characteristics of the left kidney - Size of portal vein - Size of the common bile duct - Size and characteristics of the gallbladder - Bladder volume and characteristics - Size and characteristics of the abdominal aorta - Size of the inferior vena cava in the study of a patient with hypotension  1. Describe the best thing about the ultrasound course you took 2. Please describe any suggestions for improving the ultrasound course. |
| --- |

**REFERENCES**

1. García de Casasola G, Torres Macho J. Clinical ultrasound manual. Sociedad Española de Medicina Interna. https://www.tauli.cat/hospital/images/SubSites/ServeiUrgencies/documents/PautesActuacio/Radiologia/Manual_Ecografia_clinica.pdf [Accessed 2017-11-20]
